# Dual-color space network with global priors for photo retouching

**DOI:** 10.1038/s41598-023-47186-6

**Published:** 2023-11-13

**Authors:** Pilseo Park, Heungmin Oh, Hyuncheol Kim

**Affiliations:** Pixell Bussiness Division, Pixell Lab, 4by4 Inc., 479 Gangnam-daero, Seoul, 06541 Korea

**Keywords:** Electrical and electronic engineering, Software

## Abstract

There have been growing trends using deep learning-based approaches for photo retouching which aims to enhance unattractive images and make them visually appealing. However, the existing methods only considered the RGB color space, which limited the available color information for editing. To address this issue, we propose a dual-color space network that extracts color representations from multiple color spaces to provide more robust color information. Our approach is based on the observation that converting an image to a different color space generates a new image that can be further processed by a neural network. Hence, we utilize two separate networks: a transitional network and a base network, each operating in a different color space. Specifically, the input RGB image is converted to another color space (e.g., YCbCr) using color space converter (CSC). The resulting image is then passed through the transitional network to extract color representations from the corresponding color space using color prediction module (CPM). The output of the transitional network is converted back to the RGB space and fed into the base network, which operates in RGB space. By utilizing global priors from each representation in different color spaces, we guide the retouching process to produce natural and realistic results. Experimental results demonstrate that our proposed method outperforms state-of-the-art methods on the MIT-Adobe FiveK dataset, and an in-depth analysis and ablation study highlight the advantages of our approach.

## Introduction

Digital images have become ubiquitous in our daily lives, taken with various devices such as smartphones, digital cameras, and drones, which provide significant visual information in diverse fields^1–3^. The quality of images, however, is not always optimal due to various factors, such as lighting conditions, color distortions, and camera settings. Photo retouching is a common practice to enhance the appearance of low-quality images by removing unwanted elements and improving the overall aesthetics. However, manually retouching images demands specialized expertise and training, making it difficult for everyday users to accomplish. Even for professional photo editors, retouching large batches of images can be time-consuming and monotonous. Therefore, there have been drastic demands for automated photo retouching solutions.

Recently, deep learning-based photo retouching methods have been proposed as an appropriate solution to improve image quality and visual fidelity, leveraging the power of Convolutional Neural Networks (CNNs)^4–6^. Furthermore, the pioneering study which involved gathering a vast collection of input images and their corresponding retouched images edited by experts resulted in the establishment of the MIT-Adobe FiveK dataset^7^. This has facilitated the advancement of supervised learning approaches^8,9^.

One of the challenges in deep learning-based photo retouching is how to efficiently and accurately represent the color information in an image. Generally, the most straightforward way to capture more color representation in the image required for retouching involves increasing either the number of layers in the network or the number of filters in each layer. However, previous photo retouching methods^4–6,10^ have focused such methods solely on images presented to the network in the RGB color space. Although the information obtained from the RGB space is valuable for editing the images, incorporating more diverse cues from different color spaces can facilitate the process of deep learning tasks^11,12^.

**Figure 1:**
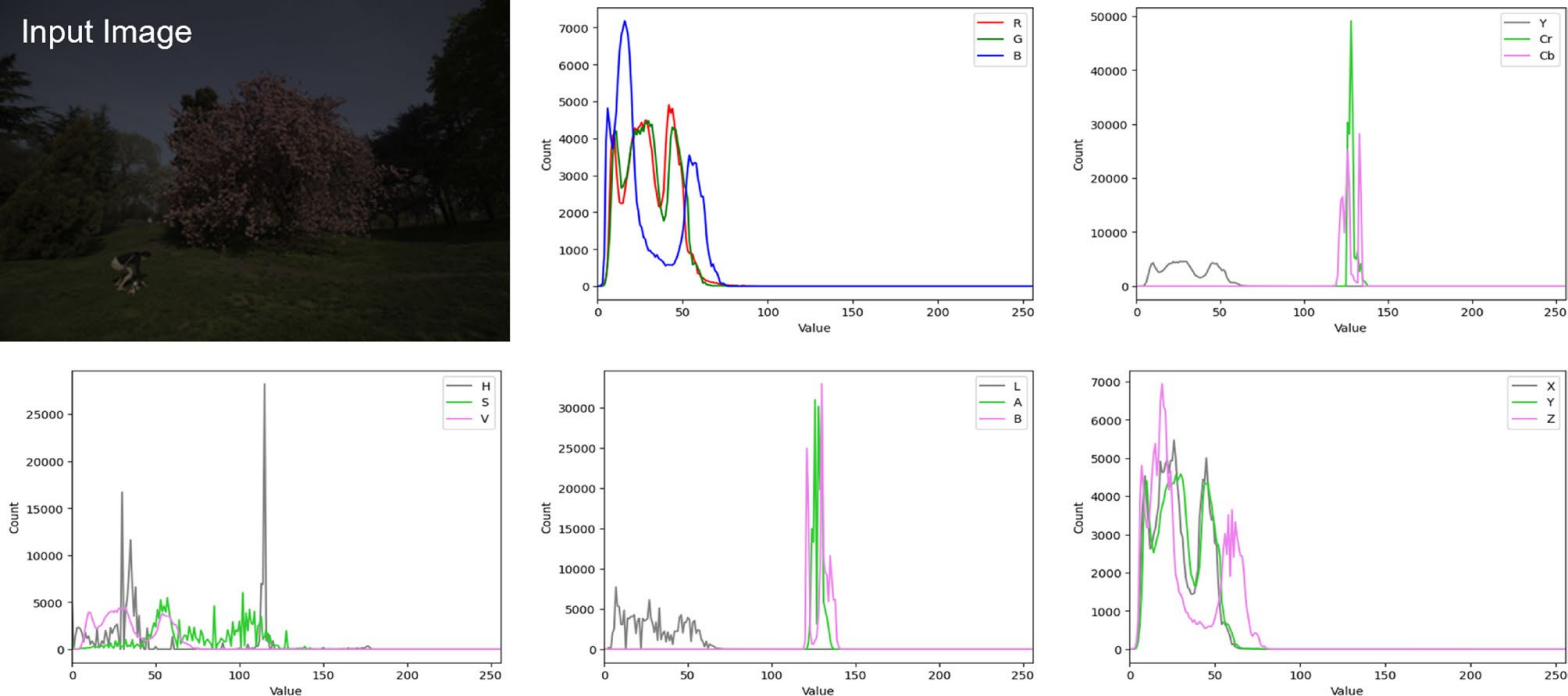
Histograms of an input image for different color spaces.

To address this issue, we aim to improve photo retouching performance by considering various color spaces. As a first step, we examined the images converted from RGB space into various alternative color spaces, such as YCbCr, HSV, LAB, and XYZ. As shown in Fig. 1, the input image show completely different histograms for each color space. In other words, transforming an image into an alternate color space results in the generation of a new image that can be effectively employed by a neural network. From this perspective, we leverage the images in multiple color spaces and their global priors to enhance the visual quality of images.

In this paper, we propose a dual-color space network that operates on two color spaces, which provides more robust color information compared to a single color space network. The network takes RGB image as input and the color representations are extracted from each color space. We obtain global priors from the each representation and utilize them to guide the retouching process towards natural and realistic results. Also, our network is designed to adopt a sequential processing framework that resembles the step-by-step workflow of humans^5,6,13^.

Our contribution can be summarized as follows:

- We introduce a dual-color space network that leverages global priors in different color spaces to enhance the overall quality of the image. Moreover, the network employs a straightforward sequential process to simplify the architecture.
- Color prediction module (CPM) and color space converter (CSC), which serve as integral components of our network, are introduced to extract features from diverse color spaces and transition between these color spaces.
- We present an extensive analysis and ablation study that highlights the benefit of the proposed method and shows intriguing properties that can guide future research directions.

## Related work

### Color space conversion

Color spaces play a crucial role in several deep learning tasks, including image classification, salient object detection, and image segmentation. Various color spaces have been used for these tasks, and several approaches have been proposed to learn the features of different color spaces. By exploiting the strengths of each color space, these approaches can improve the performance and accuracy. ColorNet^14^ proposed an architecture that can learn to classify image using different color spaces, and show that certain spaces, such as LAB and HED, can improve classification performance compared to RGB space. MCSNet^11^ transformed the images into HSV and grayscale color spaces to capture additional information on saturation and luminance. The VGG-16^15^ backbone network is then used to extract features in parallel from both the RGB channels with color information and the channels with information on saturation and luminance of the scene. Abdelsadek et al.^12^ investigated the effect of using different color spaces on image segmentation. Four different color spaces, including RGB, YCbCr, XYZ, and HSV, were compared using various image segmentation methods. These studies demonstrate that the selection of color space has a notable influence on the results.

### Color transform-based methods

The typical approach for these methods involves extracting features from a low-resolution image and then using them to predict parameters for predefined local or global color transformations. The predicted color transformation is then applied to the initial high-resolution image. Common color transformation techniques comprises of several functions such as curved-based transforms^16–18^, affine transforms^4,19,20^ and lookup tables^21,22^. The transformation functions learned by these methods can adjust to various image contents and are computationally efficient. However, their effectiveness is limited by the predetermined color transformation and may not be sufficient to accurately represent complex and non-linear color mappings between the input and retouched images.

### Sequential processing methods

These methods belong to a category that imitates the retouching workflow of humans by representing the process as a sequence of color operations. Implementing this approach is challenging as it demands additional supervision to identify the most suitable editing sequence. CSRNet^23^ explored commonly used retouching operations and showed that these operations can be expressed as multi-layer perceptrons. The layers were affected by a 32-dimensional conditional vector obtained from the input image through global feature modulation. Shi et al.^13^ proposed an operation planning algorithm to produce synthetic ground-truth sequences that can facilitate the training of the network. NeurOps^24^ replicated the conventional color operators and acquired knowledge of color transformation at the pixel level, with its intensity being determined by a scalar. These approaches do not involve converting the image into different color spaces, instead, they utilize the original RGB image for performing photo retouching.

**Figure 2:**
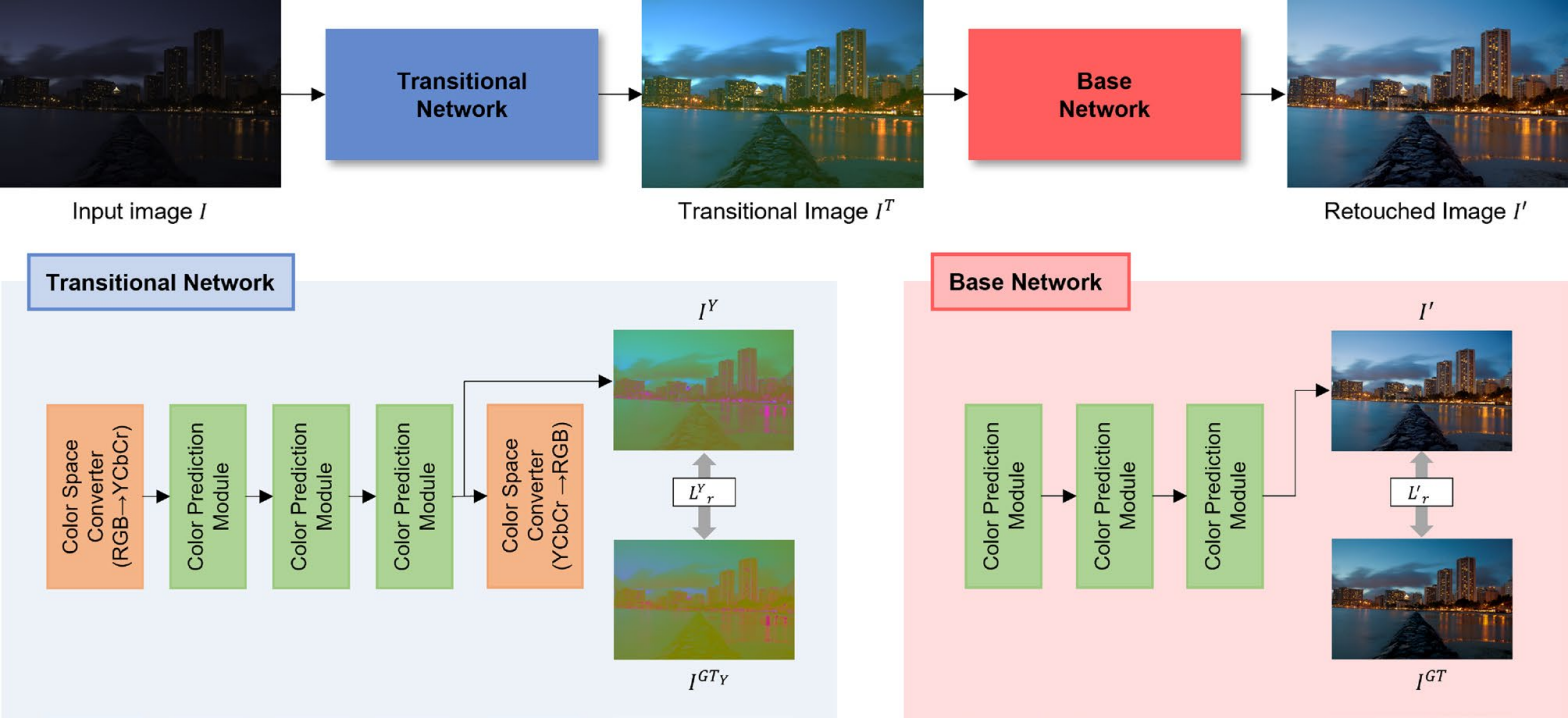
The overview of proposed dual-color space network. Our method consists of two separate networks, which operates in different color spaces.

## Method

### Dual-color space network

As shown in Fig. 2, the proposed framework includes a transitional network and a base network. The transitional network takes a low-quality image as input and produces a transitional image. Then the transitional image is fed into the base network. This structure allows the proposed method to utilize global priors from multiple spaces of a single image for enhancement.

#### Transitional network

Our study is driven by two key concepts:

1. Neural networks perceive images as numerical values. Therefore, any conversion of the color space on these images should be interpreted by the network as a completely new image.
2. Color spaces do not have a complete correlation with one another, and some images are better represented by different color spaces other than RGB space^14^.

Expanding on these concepts, two things can be concluded. Firstly, a single image can be transformed into multiple representations through color space conversion, thus achieving a similar effect to using multiple inputs. This means that we can obtain multiple global priors from a single image. Secondly, the combinations of these global priors can lead to better results. We implement the idea in the field of photo retouching by incorporating the transitional network.

Since we consider both quantitative measures and perceptual quality, we have chosen to use the YCbCr color space for the transitional network. The Y channel denotes the brightness or luminance of the image, while the Cb and Cr channels represent the chrominance. As the standard photo retouching dataset^7^ tends to feature images with an under-exposed condition, we utilize the Y channel to enhance visual results. The chrominance channels are employed together to modify color information.

As illustrated in Fig. 2(bottom left), the network consists of three CPMs and two CSCs, which will be explained carefully in Sects. 3.2 and 3.3. The network first converts the input image *I* from RGB to YCbCr. Next, the YCbCr input is processed through a series of CPMs that sequentially improve the input. Before the last CSC, the transitional YCbCr image ${I}^{Y}$ is saved to compute the reconstruction loss ${{\mathscr {L}}^{Y}}_{r}$. This approach allows the network to carry out retouching operations by using the global prior from the color space other than RGB. Finally, the network generates a transitional image ${I}^{T}$.

#### Base network

The base network takes ${I}^{T}$ as input, which is in the RGB color space. As shown in Fig. 2(bottom right), the base network is composed of three CPMs. The network utilizes the global prior from RGB space, contrary to the transitional network. The network produces the final retouched image $I'$ and computes the reconstruction loss ${\mathscr {L}'}_{r}$.

**Figure 3:**
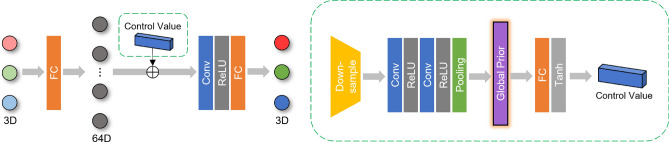
Left: The overview of color prediction module (CPM). Right: The process of obtaining the control value.

### Color prediction module (CPM)

We followed the sequential image retouching pipeline^13,23^ to build our CPM which maps each input pixel to the output pixels via pixel-wise manner. Our goal is to produce a retouched image ${I'}\in {{{\mathbb {R}}}^{{H}\times {W}\times {C}}}$ from an input image ${I}\in {{{\mathbb {R}}}^{{H}\times {W}\times {C}}}$ by implementing *N* pixel-wise mapping in a sequential manner. We set $N = 3$ for both the transitional and base network. As illustrated in Fig. 3, our CPM takes 3D image *I* as the input and generates intermediate images ${I_{n}}$:

1 $$\begin{aligned} {I_{n}} = {{CPM}_{n}}({I}_{n-1}), {1}\le {n}\le {N}, \end{aligned}$$

where ${I}_{0} = {I}$ and ${I}_{N} = {I}^{T}$ for the transitional network and ${I}_{0} = {I}^{T}$ and ${I}_{N} = {I'}$ for the base network.

Specifically, we utilize an equivariant mapping to build a simple translation^24^. As Fig. 3(left) shows, ${I}_{n-1}$ is converted into a 64D feature vector *z*. Then, we perform a straightforward translation in the feature space ${z'} = {z} + {v}$ where *v* is a control value that determines the magnitude of the translation. Lastly, the modified feature vector $z'$ is converted back to the 3D RGB space resulting in the output $I'$:

2 $$\begin{aligned} {I}_{N} = {H({G({I}_{0}) + v})}, \end{aligned}$$

where *G* and *H* denote the mappings from 3D to 64D feature vector and vice versa, respectively.

We obtain *v* as illustrated in Fig. 3(right). To incorporate global image statistics, we downsample the 3D RGB image, denoted as ${{I}^{\downarrow }}_{n-1}$, and use ${7}\times {7}$ kernel size. We represent the prediction of *v* as a mapping function denoted by *F*:

3 $$\begin{aligned} {v} = {F({{I}^{\downarrow }}_{n-1}, {d})}, {v}\in {{{\mathbb {R}}}^{d}}. \end{aligned}$$

where *d* is a feature space dimension.

*F* consists of a downsampling layer, two convolution layers, a pooling layer, and a fully connected layer. Firstly, the 3D RGB image is downsampled and the two convolutional layers are used to extract 32D feature maps. Next, three different pooling functions are utilized to determine the maximum, average, and standard deviation for each channel. These three 32D vectors are concatenated into a 96D vector, which we refer to as the global prior. As described in Sect. 3.1, our method employs the global priors from two color spaces. Finally, the fully connected layer maps the 96D global prior to a 64D control value *v*.

### Color space converter (CSC)

To obtain more comprehensive set of color information from two separate type of global priors, the proposed method utilizes both the RGB and the YCbCr space for the enhanced retouching process. RGB represents colors by combining different intensities of red, green, and blue, and is the most commonly used color space in digital images. YCbCr represents color information using Y (luminance), Cb (Chroma blue), and Cr (Chroma red).

The conversion from RGB to YCbCr color space can be represented using a conversion matrix as follows:

4 $$\begin{aligned} \begin{bmatrix} Y \\ Cb \\ Cr \end{bmatrix} = \begin{bmatrix} 0.299 &{}\quad 0.587 &{}\quad 0.114 \\ -0.169 &{}\quad -0.331 &{}\quad 0.500 \\ 0.500 &{}\quad -0.419 &{}\quad -0.081 \end{bmatrix} \cdot \begin{bmatrix} R \\ G \\ B \end{bmatrix} + \begin{bmatrix} 0 \\ 128 \\ 128 \end{bmatrix}. \end{aligned}$$

The RGB to YCbCr color transformation can also be achieved by using a conversion matrix. The matrix is as follows:

5 $$\begin{aligned} \begin{bmatrix} R \\ G \\ B \end{bmatrix} = \begin{bmatrix} 1.000 &{}\quad 0.000 &{}\quad 1.403 \\ 1.000 &{}\quad -0.344 &{}\quad -0.714 \\ 1.000 &{}\quad 1.773 &{}\quad -0.000 \end{bmatrix} \cdot \begin{bmatrix} Y \\ Cb-128 \\ Cr-128 \end{bmatrix}. \end{aligned}$$

As shown in Fig. 2(bottom left), we utilize CSCs to convert the color space between RGB and YCbCr, and implement them at the beginning and end stages of the transitional network.

### Training objective

Given an RGB image *I*, we refer to its ground truth (GT) image as ${I}^{GT}$ and the retouched image predicted by the model as $I'$. Also, referring to a YCbCr converted image ${I}^{Y}$, the GT image is denoted as ${I}^{{GT}_{Y}}$. The total loss ${{\mathscr {L}}}_{total}$ is composed of a RGB reconstruction loss ${\mathscr {L}'}_{r}$, a YCbCr reconstruction loss ${{\mathscr {L}}^{Y}}_{r}$, a total variation loss ${{\mathscr {L}}}_{tv}$, and a color loss ${{\mathscr {L}}}_{c}$.

#### Reconstruction loss

To train the model using both RGB and YCbCr color spaces, two distinct reconstruction losses are employed. Both of these losses measure the L1 difference between the predicted image and GT:

6 $$\begin{aligned}{} & {} {\mathscr {L}'}_{r} = \frac{1}{CHW}{\Vert {I'} - {I}^{GT} \Vert }_{1}, \end{aligned}$$

7 $$\begin{aligned}{} & {} {\mathscr {L}}^{Y}_{r} = \frac{1}{CHW}{\Vert {I}^{Y} - {I}^{{GT}_{Y}} \Vert }_{1}. \end{aligned}$$

#### Total variation loss

We also include total variation loss^25^ to encourage smoother and more continuous image outputs:

8 $$\begin{aligned} {\mathscr {L}}_{tv} = \frac{1}{CHW}{\Vert \nabla {I'} \Vert }_{2}, \end{aligned}$$

where $\nabla (\cdot )$ refers the gradient operator.

#### Color loss

We implement a color loss^20^ that considers RGB colors as 3D vectors and computes the angular differences between them:

9 $$\begin{aligned} {\mathscr {L}}_{c} = {1} - \frac{1}{HW} \angle ({I'},{I}^{GT}), \end{aligned}$$

where $\angle (\cdot )$ operator calculates the average cosine of the angular differences between values at each pixel.

#### Total loss function

Therefore, the complete training object of our network is:

10 $$\begin{aligned} {{\mathscr {L}}}_{total} = {\mathscr {L}'}_{r} + {\lambda }_{1}{{\mathscr {L}}^{Y}}_{r} + {\lambda }_{2}{{\mathscr {L}}}_{tv} + {\lambda }_{3}{{\mathscr {L}}}_{c} \end{aligned}$$

where ${\lambda }_{1}$, ${\lambda }_{2}$, and ${\lambda }_{3}$ are balancing hyper-parameters.

**Table 1:** Quantitative comparison with state-of-the-art methods on MIT-Adobe FiveK dataset.

Method	PSNR $\uparrow$	SSIM $\uparrow$	$\triangle {E}^{*}$ $\downarrow$	#params
White-Box^5^	18.59	0.797	13.24	8,561,762
Distort-and-Recover^6^	19.54	0.800	12.91	259,263,320
DUPE^26^	20.22	0.829	13.38	998,816
Pix2Pix^27^	22.05	0.788	11.88	11,383,427
HDRNet^4^	22.65	0.880	11.64	482,080
CSRNet^23^	23.62	0.894	10.97	36,489
NeurOp^24^	23.87	0.899	10.48	**28,108**
Ours	**24.12**	**0.900**	**10.23**	92,882

Significant values are in bold.

## Experiments

### Dataset and metrics

We conduct experiments on the MIT-Adobe FiveK dataset^7^ which is a widely-used set of raw images and corresponding retouched versions manually edited by five experts (A/B/C/D/E). We follow the common practice^4,6,26,28^, utilizing the retouched image of expert C as the GT in our experiments, and splitting training and testing sets into 4500 images and 500 images, respectively. All images are resized by reducing the longer edge to 500px while maintaining the aspect ratio.

We use PSNR^29^, SSIM^30^, and delta E ($\triangle {E}^{*}$)^31^ as metrics to evaluate the performance. $\triangle {E}^{*}$ is a color difference metric defined in the CIELAB color space and has been demonstrated to be consistent with human perception. Unlike PSNR and SSIM, a smaller $\triangle {E}^{*}$ indicates better performance.

### Implementation details

We implement our model using PyTorch framework^32^. All our experiments are conducted on a single NVIDIA RTX 3090 GPU. During training, the mini-batch size is set to 1 and run 600, 000 iterations. We use the Adam optimizer^33^ with ${\beta }_{1}$ = 0.9, ${\beta }_{2}$ = 0.99 and an initial learning rate is $5{e}^{-5}$. The weights for the balancing hyper-parameters in Eq. 10 are ${\lambda }_{1}$ = 0.01 and ${\lambda }_{2}$ = ${\lambda }_{3}$ = 0.1. The base network contains three CPMs, and the transitional network contains three CPMs and two CSCs.

### Comparisons with state-of-the-arts

We compare our model with state-of-the-art methods, including White-Box^5^, Distort-and-Recover^6^, DUPE^26^, Pix2Pix^27^, HDRNet^4^, CSRNet^23^, and NeurOp^24^ to demonstrate its effectiveness. For White-Box, Distort-and-Recover, DUPE, Pix2Pix, and HDRNet, we refer to the results from the previous work^23^. For the top two state-of-the-art methods, CSRNet and NeurOp, we retrained their models under the same experimental conditions as ours to ensure a fair comparison.

#### Quantitative comparison

The results presented in Table 1 demonstrate that our proposed model outperforms the previous state-of-the-art methods on the MIT-Adobe FiveK dataset^7^. Specifically, White-Box and Distort-and-Recover show low performance with less than 20dB in PSNR and necessitate millions of parameters. This is because they are reinforcement-learning-based methods and are not directly supervised by the GT image. One reason for this is that these methods use reinforcement learning and do not receive direct supervision from the GT image. DUPE and HDRNet exhibit fairly decent performance but require several hundred thousand parameters. Similarly, Pix2Pix performs reasonably well, but it relies on over ten million parameters.

For the top two state-of-the-art methods, CSRNet and NeurOp, our proposed model outperforms in terms of all metrics. Our model requires relatively more parameters than CSRNet and NeurOp, but less than ten thousand which is still light-weighted. The results show that the proposed method outperforms the existing methods and exhibits a lightweight architecture.

**Figure 4:**
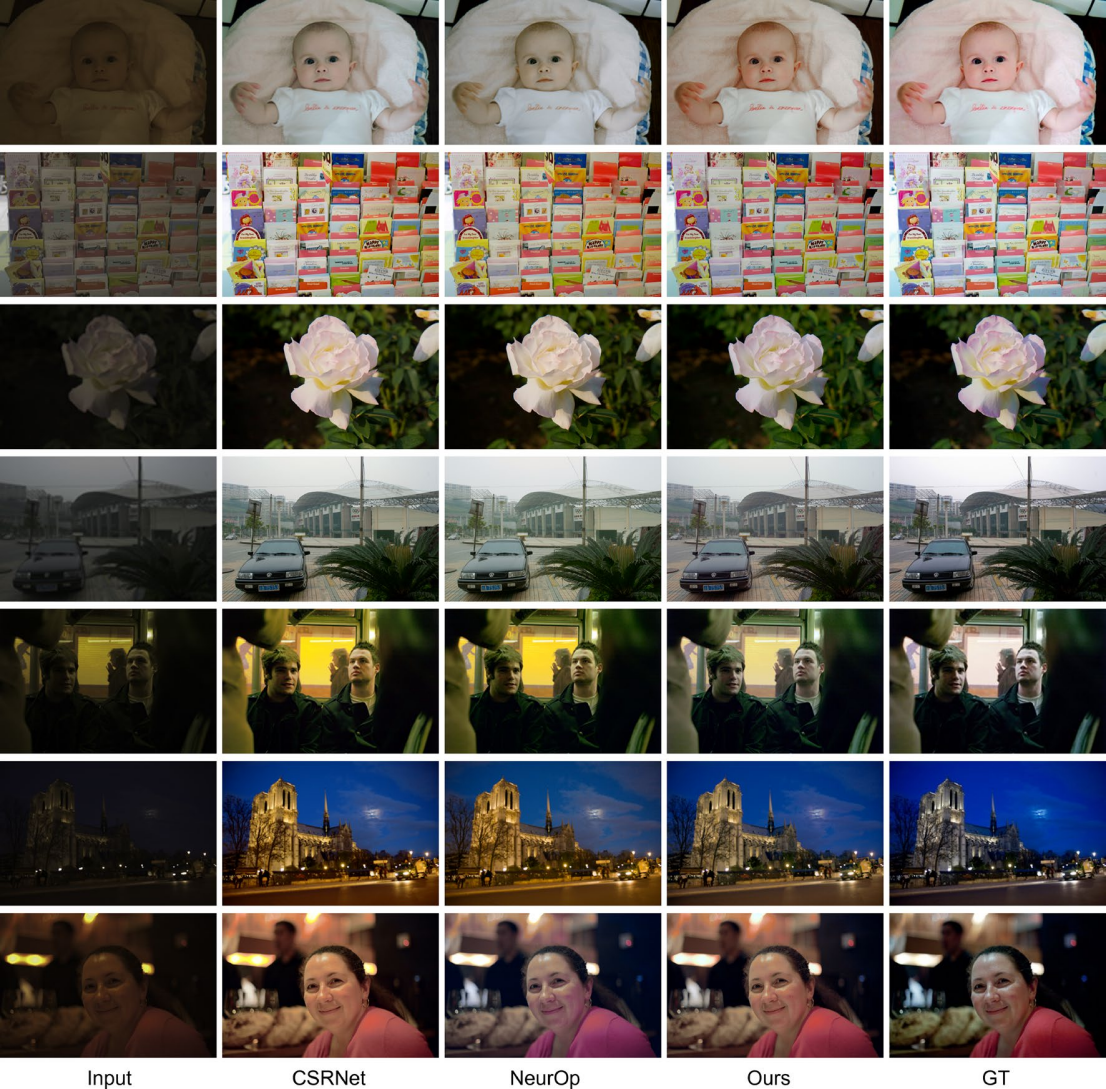
Visual comparison with state-of-the-art methods on MIT Adobe FiveK dataset. Zoom in for better visibility.

#### Visual comparison

A visual comparison with state-of-the-art methods is shown in Fig. 4. We only compared CSRNet and NeurOp, which show stable performance, as other models display poor quantitative metrics^5,6,26^, contain unpleasing artifacts^27^, or produce images with unrealistic color in some areas^4^. Compared with these two models, the retouched images demonstrate the effectiveness of our method. Specifically, the first, second, and third row shows that our method can enhance the input image vividly and naturally. For the fourth, fifth, and sixth rows, the results obtained from our method show the most realistic images that resemble the GT images. The seventh row of the human photo has a lower resemblance between all three methods and the GT images, but our results demonstrate the most realistic natural skin color and fewer color shifts.

**Figure 5:**
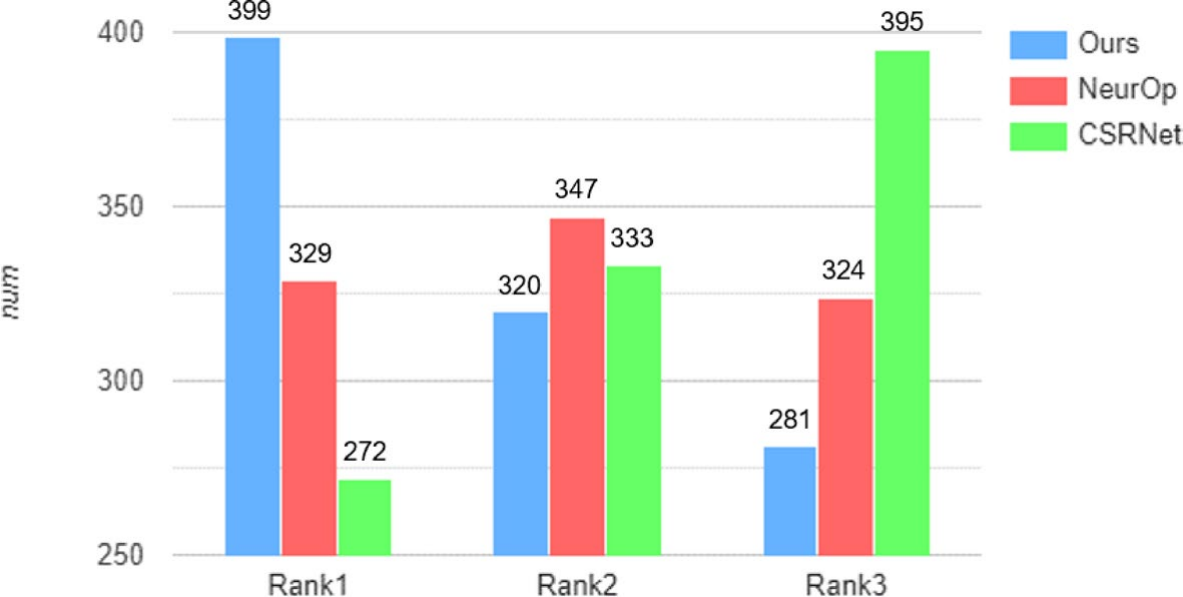
MOS test ranking results. Rank 1 most closely represents the GT image and indicates the results most preferred by the participants..

#### User study

We have conducted a Mean Opinion Score (MOS) test to present a user study. We selected a total of 20 participants and randomly chose 50 images from the test set for each of them. Participants were asked to rank the retouched results from three versions, CSRNet^23^, NeurOp^24^, and ours based on their similarity to the GT image and visual appeal, assigning them 1st, 2nd, and 3rd place rankings. As shown in Fig. 5, our results achieve better visual ranking against CSRNet and NeurOp with 399 images ranked first and 281 images ranked third. These results indicate that our retouched results are visually more favorable to the participants compared to other methods.

**Table 2:** Evaluation of PSNR and SSIM on the Y channel of YCbCr space using the MIT-Adobe FiveK dataset.

Method	PSNR$\uparrow$	SSIM$\uparrow$
CSRNet^23^	26.05	0.939
NeurOp^24^	26.36	0.940
Ours	**26.58**	**0.942**

Significant values are in bold.

### Ability to capture luminance

**Figure 6:**
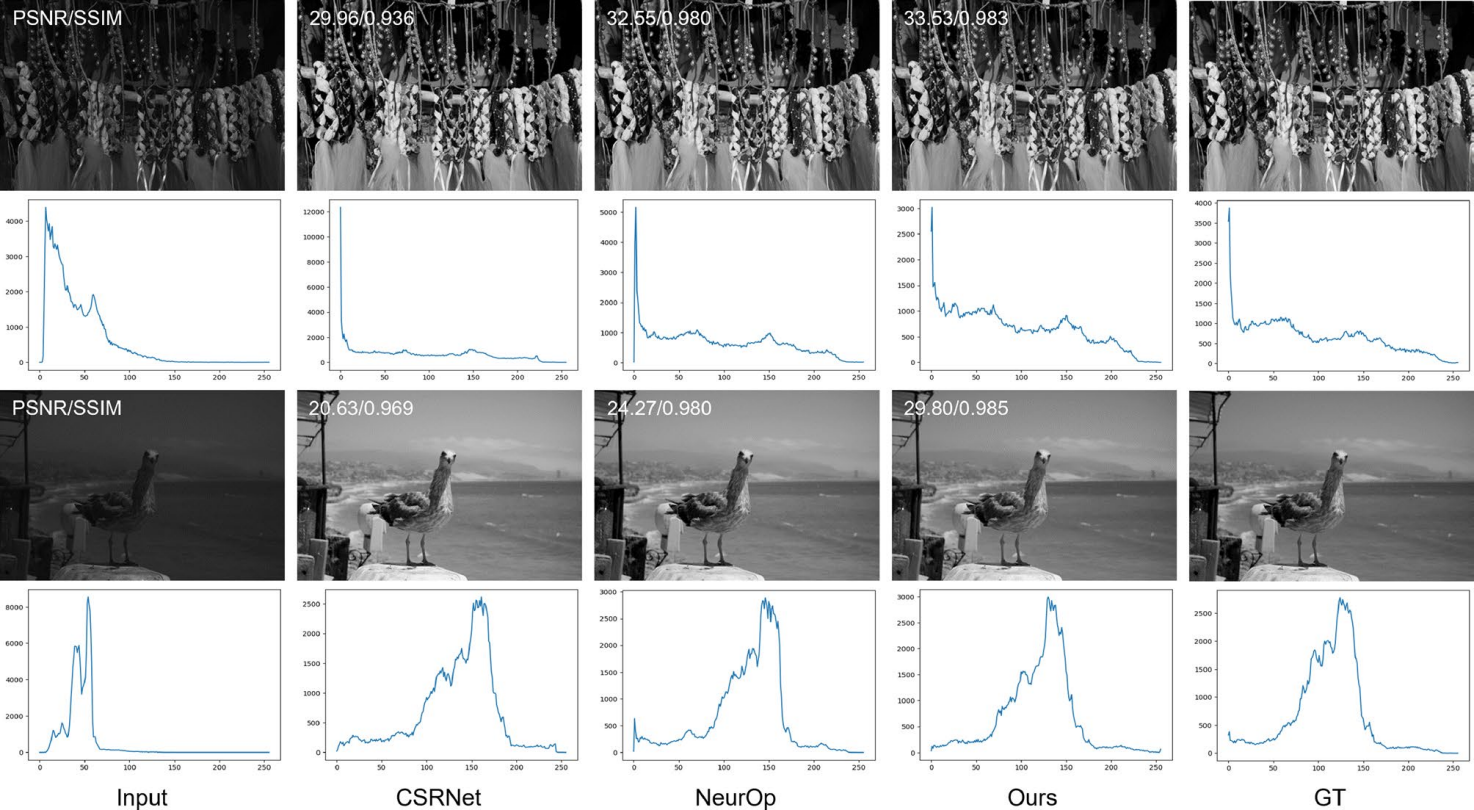
Visual comparison on the Y channel of YCbCr space using the MIT-Adobe FiveK dataset. For better understanding, PSNR values and histograms are provided.

Since we used the YCbCr color space in our transitional network to utilize the luminance feature, we have presented quantitative and qualitative results for the Y channel. Table 2 demonstrates that our proposed method outperforms existing methods in both PSNR and SSIM on the Y channel. Specifically, our model achieves a higher PSNR/SSIM than CSRNet by 0.53db/0.003 and NeurOp by 0.22db/0.002. Additionally, Fig. 6 illustrates that our results show the highest PSNR/SSIM values and their histograms closely resemble those of the GT images. These results indicate that our method effectively extracts the luminance feature from the GT image and utilizes it to generate the final retouched image.

### Ablation study

**Figure 7:**
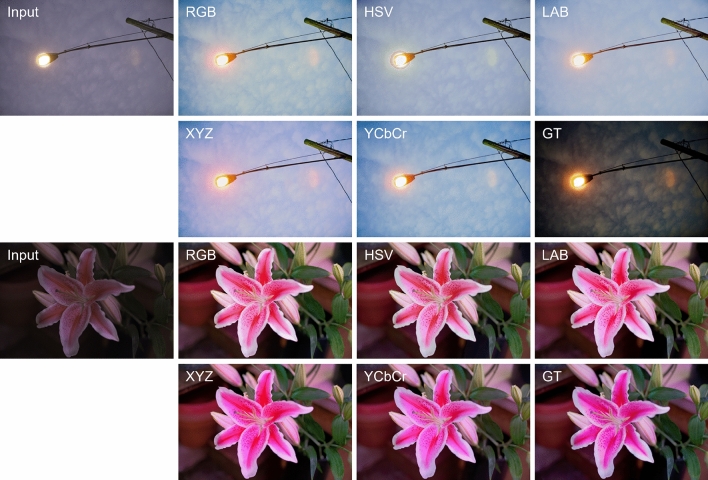
Visual comparison for the different color spaces of the transitional network on the MIT-Adobe FiveK dataset. Zoom in for better visibility.

To validate the choice of YCbCr color space in the transitional network, we conducted ablation studies by training the model using three additional color spaces, HSV, LAB, and XYZ, and comparing their performance. For a fair comparison in the RGB space, we employed the same process as in other color spaces. The result was obtained using the entire network, including both the base network and transitional network from which CSCs were removed, to maintain the same number of parameters. The quantitative results, shown in Table 3, demonstrate that using multiple color spaces is generally more effective then using just one RGB color space. The LAB color space, which is utilized by the $\triangle {E}^{*}$ metric, showed the best performance in terms of $\triangle {E}^{*}$. In addition, we present visual comparison results in Fig. 7. All models using the different color spaces generated pleasing results without any artifacts or unnaturalness. However, our retouched images produces the most vivid and closest-to-GT results. The results suggest that it is possible to conduct further research by exploring different color spaces and various combinations of them.

**Table 3:** Quantitative comparison for different color spaces of the transitional network on MIT-Adobe FiveK dataset.

Color space	PSNR$\uparrow$	SSIM$\uparrow$	$\triangle {E}^{*}\downarrow$
HSV	23.69	0.892	10.32
RGB	23.87	0.900	10.30
LAB	23.99	0.900	**10.15**
XYZ	24.00	**0.901**	10.30
YCbCr (Ours)	**24.12**	0.900	10.23

Significant values are in bold.

## Conclusion

This paper introduces a novel dual-color space network that provides robust color information by operating on two distinct color spaces, surpassing the capabilities of a single-color space network. By employing a transitional network and a base network, color representation is extracted from both color spaces. This approach allows the proposed network to incorporate global priors from both color spaces, guiding the retouching process toward producing natural and realistic results. Our experiments demonstrated that the proposed method achieves higher accuracy and generates retouched images that are more natural and visually striking compared to existing state-of-the-art methods. Our future work aims to investigate alternative color spaces and explore different combinations of them to further enhance the modeling capabilities of the network.

While our approach yields promising results, there are still limitations that need addressing. Although our proposed method outperforms previous methods across all metrics and retains a lightweight model, the process involved in converting between color spaces could elevate computational costs for high-resolution images. In addition, CPMs and CSCs in the transitional network primarily operate in the YCbCr color space, emphasizing the Y channel for luminance capture. Our choice was influenced by the commonly used MIT-Adobe FiveK dataset which contains under-exposed images. For an exceptional test set case that is relatively less low-exposure and GT image contains overall dark pixels, the results deviate from the GT images although the result is realistic and aesthetically pleasing. We hope that future research will introduce its practical application in real-world situations with various image conditions.
